# Use of reclaimed urban wastewater for the production of hydroponic barley forage: water characteristics, feed quality and effects on health status and production of lactating cows

**DOI:** 10.3389/fvets.2023.1274466

**Published:** 2023-11-17

**Authors:** Luigi Ceci, Maria Alfonsa Cavalera, Francesco Serrapica, Antonio Di Francia, Felicia Masucci, Grazia Carelli

**Affiliations:** ^1^Department of Veterinary Medicine, University of Bari, Valenzano, Italy; ^2^Department of Agricultural Sciences, University of Naples Federico II, Portici, Italy

**Keywords:** hydroponic barley forage, reclaimed urban wastewater, dairy cows, hematochemical profile, rumen pH, milk traits, digestibility, nitrogen metabolism

## Abstract

The safety of reclaimed urban wastewater (RUW) for the production of hydroponic barley forage (HBF) was evaluated in terms of effluent and forage characteristics, as well as the health and performance of lactating cows. The study was conducted on a dairy farm equipped with two hydroponic chambers producing approximately 620 kg/d of HBF as fed. For experimental purposes, HBF was produced using RUW collected from an aqueduct plant processing urban wastewater in a membrane bioreactor treatment chain. A feeding trial was carried out with HBF derived from RUW. Sixty lactating cows were randomly assigned to two balanced groups fed a standard total mixed ration (TMR) or a TMR in which 10 kg of HBF replaced 1 kg of oat hay and 0.5 kg of maize. The experimental period lasted 7 weeks, including a 2-week adaptation period, during which each cow underwent a physical examination, BCS scoring, blood sampling for a complete blood count and biochemical panel, recording of body weight and milk yield and quality, including fatty acid composition and heavy metal content. Ruminal pH was continuously monitored by reticulorumen boluses, and nutrient digestibility and N balance were determined at week 7. RUW showed an acceptable microbial load and an overall good quality as irrigation water, even though the supply of N and P did not influence the yield and quality of HBF. The characteristics of HBF reflected the quality of RUW supplied to the hydroponic chambers and no anomalous components (i.e., high ion concentration) were found. Feeding RW-derived HBF to lactating cows had no major positive or negative effects on animal health and production, including milk quality, ruminal pH, *in vivo* digestibility, and N balance. The use of RUW under the conditions tested appears to be safe for the health status of lactating cows and the quality of the milk obtained. Overall, the results do not reveal any major limitations for the use of tertiary wastewater as irrigation water for the hydroponic production of forage barley, so that a wider use of wastewater in hydroponic systems seems realistic.

## Graphical Abstract

Created with BioRender.com. 
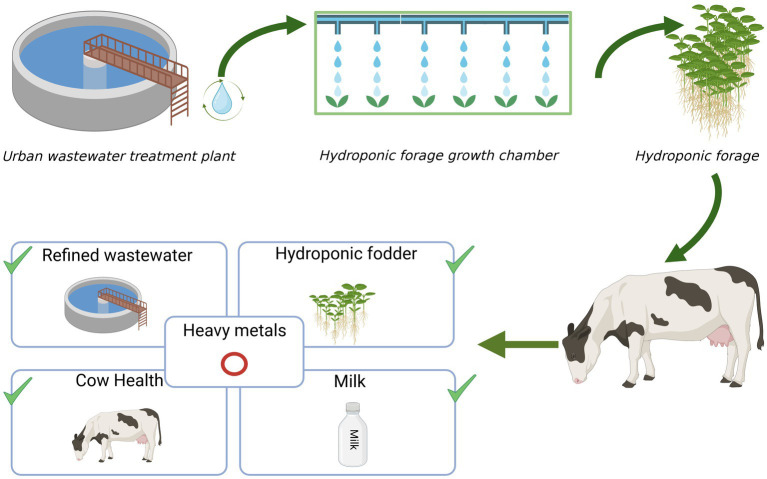

## 1 Introduction

Ongoing climate change threatens global freshwater security which is one of the United Nations sustainable development goals (1). Despite the inevitable uncertainty in forecasting models, the countries of the Southern European Union (EU) are expected to experience severe changes in both the availability and quality of water resources, which potentially lead to unprecedented pressure on the water resources (2). Currently, the agricultural sector of some Southern regions of the Euro-Mediterranean area already uses 80% of water resources, while the rest of Europe consumes only 44% and the world about 75%, on average (3, 4). Within the primary sector, it is estimated that livestock production uses 29% of the total agriculture water demand, with a large going to crops to produce animal feed (5). In this scenario, recovering urban treated or reclaimed wastewater for irrigation purposes has an enormous potential to face increasing water scarcity, preserving potable resources and reducing the environmental impact associated with the discharge of effluents into water bodies (6, 7). To date, the use of reclaimed wastewater in producing vegetables and fruit for human consumption has been successfully implemented in many rural Mediterranean areas, proving an economically feasible option to mitigate the water consumption associated with several crops production (8–11). However, less attention has been paid to the use of wastewater in forage and feed production systems (5, 12).

The use of wastewater for agricultural purposes presents concerns related to the presence of pathogenic microorganisms that could be a source of infection for humans and animals (5, 13). Similarly, there may be organic and inorganic chemicals that can enter the food chain, accumulate in the soil, be absorbed by plants, and then have humans as final consumers (14, 15). Currently, in the European context, the use of wastewater for irrigation is strictly regulated. In treatment plants, urban wastewater, as defined by EU Directive 91/271, undergoes sequential treatment to remove suspended solids and pollutants as well as unwanted compounds and microorganisms (16). Wastewater treated to remove pathogens and stabilize water quality, hereafter referred to as reclaimed urban wastewater (RUW), can be used for irrigation of raw and processed food crops and non-food crops, such as pasture and forage (17). In this scenario, hydroponics is now seen as an agricultural technology that can be easily adapted to reuse alternative water sources, reducing both irrigation water demand and wastewater disposal, and improving the environmental and economic sustainability of crop production and wastewater treatment technologies (18). Hydroponics is a type of vertical, soilless, indoor cultivation in which the growing environment is controlled in terms of temperature, humidity, light and in which the use of chemicals tends to be much lower than in traditional crop systems (19, 20). In recent years, hydroponics has also been proposed to produce fresh forage, a natural, palatable, and easily digestible feed that can improve the health and performance of lactating animals and even the nutraceutical properties of dairy products (21–23), which is however not available year-round in the climatic conditions of southern Europe (24, 25). Fresh hydroponic forage (HF) production is based on indoor germination and growth for a short time (6–8 d) of cereal seeds (especially barley) characterized by high and rapid germinability (26). In addition to producing fresh, high-quality forage all year round, regardless of weather conditions and without land requirements, HF production is also expected to reduce water consumption compared to conventional forage crops (27, 28) and has been proposed as a suitable system for livestock farms with limited availability of arable land and irrigation water (29, 30). The use of alternative water sources has been found to increase water use efficiency as well as the nutritional quality of HF (31–34), but the effects of such forage on animal performance and health have not been addressed so far.

Furthermore, in the European context, taking into consideration the requirements defined by the recently enacted European regulation 2020/741 (17), it is pivotal to assess the potential animal health risks associated with the reuse of RUW for hydroponic fodder production. Indeed, though hydroponic fodder is not explicitly mentioned in the EU regulation, it falls under the category “non-food crops,” including pasture and forage.

Thus, assuming its safety, this study was designed as a first attempt to evaluate the use of RUW for HBF production in terms of water characteristics, feed quality, and effects on the health and production of dairy cows, elements that have been considered separately or not at all in the available literature.

## 2 Materials and methods

### 2.1 Ethics approval

The study was conducted according to the guidelines of the Declaration of Helsinki and approved by the Institutional Review Board of the Italian Ministry of Health (protocol code 74371-X/10, n. 1,031/2020-PR, Date of approval 19, November 2021). The withdrawal and use of RUW were authorized by the Apulian Aqueduct (Acquedotto Pugliese S.p.A.) which was a partner in the framework of the Hydrofodderpuglia research project financed by the Apulian region (PO FE5R 2014/2020-Azione 6.4-Sub-Az 6.4.a.DGR 2321/2017).

### 2.2 Study site and production of hydroponic barley fodder

The study was carried out on a dairy farm in the Apulian region, in southern Italy (40°48′N 16°56′E; 360 m a.s.l.). The farm has two hydroponics chambers (E-6-TC and EC-2-T models, Eleusis International, Madrid, Spain) with a potential daily production of hydroponic barley fodder (HBF) of 500 kg and 120 kg and a capacity of 32 and 8 perforated polyethylene trays (60×60 cm), respectively. Regardless of production capacity, each chamber consisted of a well-insulated room equipped with climate control, air extractors, a surface irrigation watering system, a fluorescent tube lighting system (40 W, with 12 to 16 daily light), and a set of shelves where the seed germination trays are placed. The temperature inside the chambers was set to a working range of 18°C to 21°C and the relative humidity was adjusted by about 70% using air circulation. Clean, sound, intact, untreated, barley seeds (*Hordeum vulgare* L.) of high quality (germination above 95%) were used. Daily, the seeds were first soaked in 1.5% sodium hypochlorite solution for 30 min, rinsed, and soaked in fresh water for 16 h. Then, the water was drained, and the seeds were left without water for 24 h (pre-germination). Subsequently, the seeds were spread into the perforated polyethylene trays at a sowing rate of 4–6 kg/m2. The growth cycle, from seed placement to harvest lasted 8 days, after which the fodder, consisting of a mass of roots, seed kernels and the aerial green part of the seedlings (from 12 to 14 cm), was manually discharged from the trays, scored for the presence of mould according to the five-point scale of Soder et al. (35), and loaded into the mixer wagon equipped with an electronic scale for inclusion in the total mixed ration (TMR).

For experimental purposes, HBF was produced using RUW taken from the treatment plant of the Apulian aqueduct located in Noci (40°48′N 17°08′E), which adopts a membrane bio reactor wastewater treatment chain. Once a week, RUW was taken from the waterworks reservoirs, transported to the farms in a dedicated tank and stored in non-toxic polyethylene tanks with the weekly addition of chlorine dioxide (0.58 ppm/L), connected to the growth chamber irrigation system. The amount of water required to daily produce 620 kg of HBF was approximately 1.5 m^3^. Before the start of the feeding trial, the characteristics of RUW were assessed for the main water quality parameters (17, 36) by a reference laboratory (EuroQuality Lab S.r.l., Gioia del Colle, Italy) according to standard methods (Supplementary Table S1).

To evaluate the potential influence of RUW on forage growth, the yield and height of forage produced using well water and RUW were measured in October 2021. Measurements were taken in both chambers for 10 consecutive days for each type of water. Furthermore, two samples of the forages so produced were evaluated for the presence and load of *Escherichia coli* (β-glucuronidase method) and *Salmonella* spp. and for the content of metals and non-metals (N, P, Na, Cd, Cr, Cu, Mn, Ni, Pb, Zn, Fe, K, Ca, Mg) by a reference laboratory (EuroQuality Lab S.r.l., Gioia del Colle, Italy) according to standard methods (Supplementary Table S2).

### 2.3 Animal enrolment

In November 2021, the health status of eighty lactating cows from the herd was evaluated. The animals were visually examined, weighed, and scored for body condition using a 1 to 5 scale (37). Blood samples were taken from the coccygeal vein, immediately used for quantification of the beta-hydroxybutyrate (BHB) level using a ketometer (CentriVet GK), and placed in a K3 EDTA tube (10 mL) to perform a complete blood count (CBC) (CELL-DYN 3700 Hematology Analyzer, Abbott), and in a plain tube (5 mL) to obtain serum after centrifugation (15 min at 1500 × g) for the biochemical panel (including albumin (Alb), alkaline phosphatase (ALP), alanine transaminase (ALT), aspartate transaminase (AST), calcium (Ca), chloride (Cl), creatinine (Cr), glucose (Glu), potassium (K), magnesium (Mg), sodium (Na), not esterified fatty acids (NEFA), phosphorus (PHOS), total bilirubin (T Bil), total iron (Ti), total proteins (Tp); Beckman Coulter, Clinical Chemistry Analyzer AU680). Furthermore, faecal samples (about 10 g) were collected from the rectum by using sterile polyethylene gloves, stored in plastic bags, and examined for gastrointestinal helminths (38). Deworming with eprinomectin (Eprinex Multiple Pour, Boehringer Ingelheim) was performed due to positive results. To be enrolled in the study, cows had to be clinically healthy, have a temperature of 38.8°C, 5–6 rumen contractions every three minutes measured by auscultation of the lumbar fossa, and no clinical signs of laminitis, metritis, and mastitis.

### 2.4 Study design, animals, and diets

**Figure 1:**
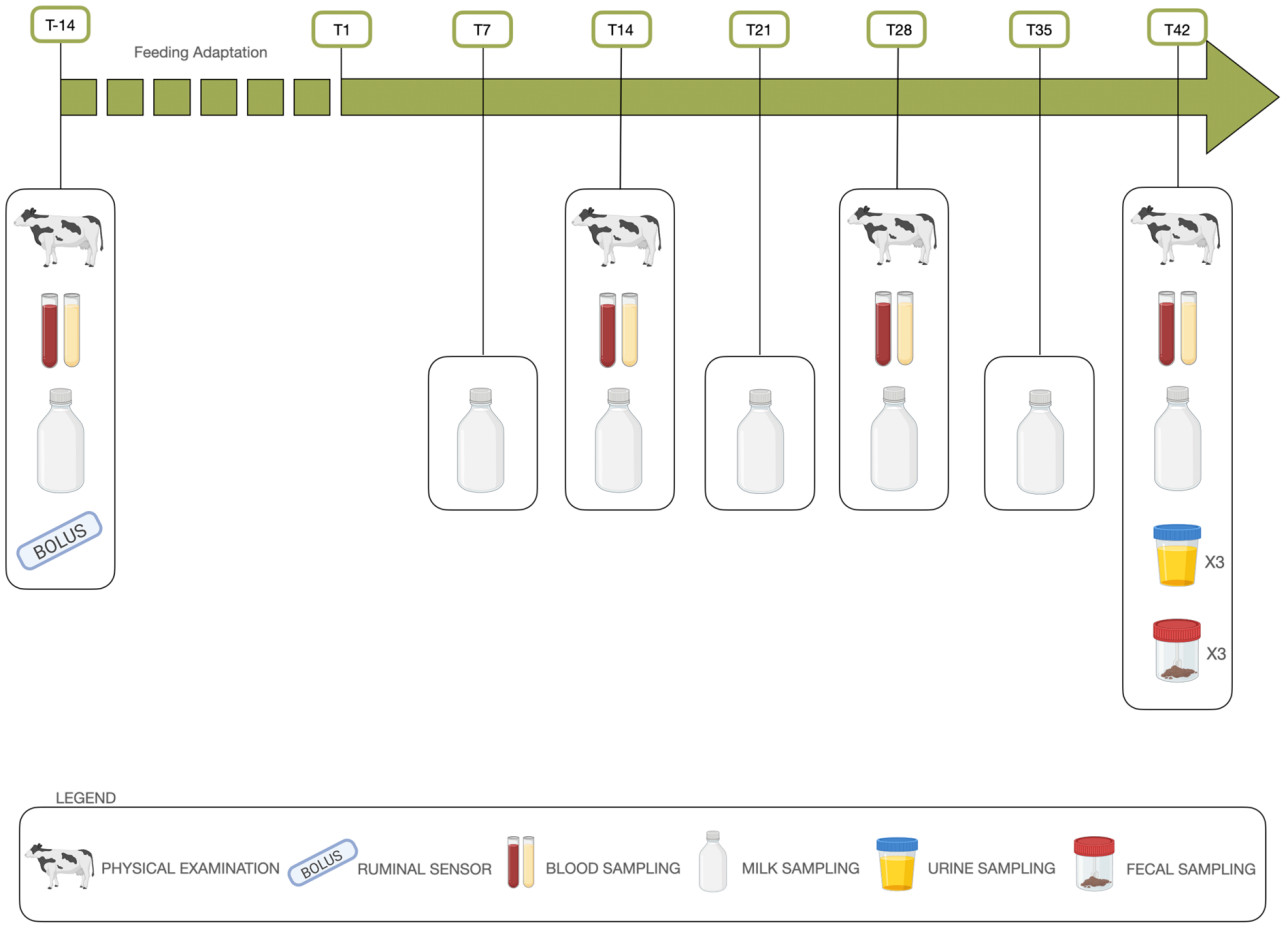
Timeline of the study project including time points and scheduled procedures. Created with BioRender.com.

Sixty dairy cows matched the selection criteria and were enrolled in a prospective, randomized, controlled, on field study that lasted from December 2021 to February 2022. The enrolled cows were randomized into two groups balanced for breed, milk yield, days in milk (DIM), parity, body condition score (BCS), and body weight (BW) (Supplementary Table S3). The control group (CG) was fed the TMR used by the farmer, while the other group (HBFG) was fed the same TMR in which 10 kg of HBF replaced 1 kg of oat hay, and 0.5 kg of maize. The diets were isonitrogenous and isoenergetic and formulated for a production level of 28 kg of energy-correct milk (ECM) per day (39). Rations were fed twice a day (08:00 and 15:00) in equal amounts, with several pushing of feed to the animals. Feed refusals (5%–10%) were removed daily before discharging the freshly prepared TMR. Animals had free access to drinking water. The groups were housed in two adjacent concrete-floored barns equipped with deep-bedded stalls (2.6 × 1.25 m, sand bedding) and an open-access clay training field (30 × 10 m). The trial lasted 9 weeks, 2 weeks for adaptation to diets, and 7 for experimental measurements and sampling according to the schedule shown in Figure 1.

### 2.5 Physical examination and blood sampling

The physical examination, body weight recording, BCS scoring, and blood sampling of the enrolled cows were repeated at T-14, T14, T28, and T42 as described above (Figure 1).

### 2.6 Dry matter intake, feed sorting, and feed analyses

Feed intake was weekly measured on a group basis by the difference between TMR discharged and residuals. Feeds and TMR were also sampled, pooled by group, and analyzed to determine the content of dry matter (DM; method 930.15), ash (method 942.05), crude protein (CP; method 976.05), and ether extract (EE; method 954.02) (40). Neutral detergent fiber (NDF), acid detergent fiber (ADF) inclusive of residual ash (41), acid detergent lignin (ADL) (42), total sugar (43), and enzymatic starch (44) were also determined. Proteins were fractionated (PA, PB1, PB2, PB3, PC) according to the Cornell Net Carbohydrate and Protein System (CNCPS) version 6.5 (45). The non-fibrous carbohydrates (NFC) were calculated as detailed elsewhere (46, 47). Additional samples of TMRs and refusals were taken every two weeks during the sampling period to assess physical structure of the diets (particle size, and physically effective NDF) and sorting activity by the cows. The particle size distribution of the fresh TMRs and refusals was stratified into long (>19 mm), medium (<19 mm, >8 mm), and short (<8 mm; Pan) particles using a Penn State particle separator (PSPS) with two screens (19 and 8 mm) (48). The physical effectiveness factor (pef), physically effective NDF (peNDF), and feed sorting index (SI) of the diets were determined according to DeVries et al. (49).

### 2.7 Milk sampling and analysis

Milk yield of each cow was measured using an electronic machine recorder (DeLaval Corp., Tumba, Sweden) and sampled in the morning milking (07:00) at weekly intervals (Figure 1). The samples were sent refrigerated at 4°C to the laboratory for the analysis of fat, protein, casein, lactose, total solids, non-fat solids, milk urea nitrogen (MUN), cryoscopy index, titration acidity, somatic cell count (SCC) and milk clotting properties which were performed on the same day using an infrared milk analyzer (CombiFoss TM7, Foss, Hillerød, Denmark). At T42, two more milk samples were collected from a selected subgroup of 10 cows/diet. One was evaluated for heavy metal concentration (As, Cd, Co, Cu, Ni, Pb) by inductively coupled plasma mass spectrometry (ICP-MS) while the other was used to determine fatty acid (FA) composition as detailed elsewhere (50). Milk fat was extracted by the Röse-Gottlieb method, the Supelco 37 component mixture (Supelco, Bellefonte, PA, United States) and a mixture of conjugated linoleic acid isomers (Nu-Chek Prep. Inc. Elysian, MN, United States) were used as external standards for gas chromatographic analysis.

### 2.8 Monitoring of ruminal pH

At enrolment (Figure 1), all cows received an indwelling wireless reticolo-ruminal sensor (pH Plus Bolus; smaXtec Animal Care GmbH, Graz, Austria) according to the manufacturer’s directions. Boluses provided continuous measurement of rumen pH, body temperature and activity levels of animals. The data were recorded every 10 min, transmitted wirelessly to a base station placed in the barn, and available in real time from a computer software/cell phone application. The risk of subacute rumen acidosis (SARA) was notified by the ruminal bolus when the pH value fell below 5.8. The daily pH mean, minimum and maximum values were calculated per cow and per day. The percentage of animals that presented at least one SARA risk alarm, number, and time (min) of SARA risk alarms were also measured.

### 2.9 Total tract apparent digestibility, microbial protein synthesis, and nitrogen balance

At T42 and for 3 consecutive days, faecal grab samples (∼200 g) were collected three times daily from a selected subgroup of 10 cows/diet, kept at +4°C in closed plastic bags, and then composited by cow. Samples of faeces, diets and refusals were dried, ground and analyzed for acid insoluble ash (AIA) (51), as well as for ash, organic matter (OM), CP, and NDF. The AIA content was used as an internal marker to estimate the apparent total tract digestibility of nutrients and the fecal output of DM (52).

At the same time as the faecal sampling, spot urine samples were also gathered either through voluntary urination or stimulation of the pudendal nerve according to the procedure of Spanghero (53). The samples (∼100 mL) were immediately acidified (10% v/v with sulfuric acid solution) and stored at −20° C until analysis. After thawing (4° C), the samples were assessed for total N (40) and creatinine and purine derivatives (PD; i.e., allantoin and uric acid) as described by George et al. (54). Daily urine volume and excretion of total N, allantoin, uric acid, and total PD (allantoin plus uric acid) were estimated based on BW and urinary creatinine concentration assuming a constant creatinine excretion rate of 29 mg/kg of BW (55). The ruminal synthesis of microbial nitrogen (MN) was estimated from the excretion of PD using the equation of Chen and Gomez (56). Nitrogen balance (NB) was calculated as reported by Spanghero and Kowalski (57).

### 2.10 Statistical analysis

The *a priori* sample size was based on 30 as a minimum and the ratio of 1:1 sample size was chosen. Bovine characteristics are reported as mean ± standard deviation (M ± SD), and as frequencies and percentages (%) for categorical variables. For testing the associations and the corrected randomization between arms (HBFG vs CG), the Wilcoxon rank-sum (Mann–Whitney) for continuous, and Chi-square or proportion test for categorical were used. Before statistical analysis, normality and homogeneity of variance of data were tested by the tests of Shapiro-Wilks and Levene, respectively. Repeated measures ANOVA was used to determine the mean difference of repeated parameters (namely BW, BCS, milk production, biochemical panel, and CBC) with treatment, time, and interaction as factors. Data on dry matter intake, *in vivo* digestibility, and nitrogen balance were analyzed by one-way ANOVA to determine the fixed effects of the treatment. Rumen bolus data were analyzed using the Wilcoxon rank sum test (Mann–Whitney). When testing the null hypothesis of no association, the probability level of error at two tails was 0.05. All statistical computations were made using StataCorp. 2021. Stata Statistical Software: Release 17. College Station, TX: StataCorp LLC.

## 3 Results

### 3.1 Reclaimed urban wastewater and hydroponic forages findings

The microbiological and chemical characteristics of the RUW used for HBF production are shown in Tables 1, 2. To assess the irrigation quality of RUW, the guidelines reviewed by Hashem and Qi (14) and Ayers and Westcot (36) were used for the parameters not included in Regulation 2020/741 (17).

**Table 1:** Characteristics of the reclaimed urban wastewater used in the study in relation to the EU requirements for irrigation use (regulation 2020/741).

Parameter	Reclaimed urban wastewater	Classification^1^
Turbidity, NTU	<1	A
Biochemical oxygen demand (BOD), mg O_2_/L	10.7	B
Total suspended solids, mg/L	<1	A
*Escherichia coli*, CFU/100 mL	55	B
Intestinal nematodes, eggs/L	<1	A

^1^ Class A, RUW suited for all food crops, including root crops consumed raw and food crops where the edible portion is in direct contact with reclaimed water. Class B, RUW suited for non-food crops including crops to feed milk- or meat-producing animals.

**Table 2:** *Salmonella* load and chemical and physical characteristics of the reclaimed urban wastewater used and recommended range for irrigation use.

Parameter	Reclaimed urban wastewater	Recommended range
*Salmonella* spp., CFU/g	No detected	0 ^a^
pH	7.54	6.5–8.4^a^
Electrical conductivity at 20°C, μS/cm	845	<700^a^
Total dissolved solids 180°C, mg/L	432	<1,000^b^
Sodium adsorption ratio (SAR), meq/L	3.9	3÷9^b^
Ammonium Nitrogen, mg/L N-NH_4_	1.2	<15 ^a^
Nitrate Nitrogen, mg/L N-NO_3_	5.1	<5.0^a^
Total Nitrogen, mg/L N	9.1	<5.0 ^a^
Phosphorus, mg/L P	1.7	<2.0 ^a^
Chlorine, mg/L Cl	149	<100^a^
Fluoride, mg/L F	0.22	1÷15^b^
Sodium, mg/L Na	138	<70 ^a^
Potassium, mg/L K	19.1	–
Magnesium, mg/L Mg	10.9	–
Calcium, mg/L Ca	77.6	–
Aluminium, μg/L Al	0.039	5,000÷20000^b^
Arsenic, μg/L As	0.005	100÷2000^b^
Boron, μg/L B	0.1888	750÷2000^b^
Cadmium, μg/L Cd	0.005	10÷50^b^
Chromium, μg/L Cr	0.005	100÷1000^b^
Iron, μg/L Fe	0.031	5,000÷20000^b^
Manganese, μg/L Mn	0.0095	200÷10000^b^
Mercury, μg/L Hg	0.0005	<1 ^b^
Nickel, μg/L Ni	0.005	200÷2000^b^
Lead, μg/L Pb	0.005	5,000÷10000^b^
Copper, μg/L Cu	0.005	200÷5000^b^
Vanadium, μg/L Va	0.005	100÷ 1000^b^
Zinc, μg/L Zn	0.319	2000÷10000^b^

a According to Ayers and Westcot (36).

b Optimal value for sprinkler irrigation suggested by Hashem and Qi (14).

The RUW was classified in class A for turbidity, total suspended solids, and presence of nematode eggs, whereas the BOD value and *E. coli* load led to the wastewater being classified as Class B (17). No *Salmonella* spp. was not detected. Levels above the recommended range but far from dangerous levels were detected for electrical conductibility, NO^−3^, total N, Na and Cl. The levels of metals were below or just close to detection limits. Table 3 shows the contaminant levels of HBF produced with RUW compared to the reference sample produced with well water.

**Table 3:** Load of *Escherichia coli* and *Salmonella* spp. and content of metals and non-metals of hydroponic barley forage (HBF) produced with reclaimed wastewater and with well water.

Parameter	HBF produced with reclaimed wastewater	HBF produced with well water
*Escherichia coli*, CFU/g	<10	<10
*Salmonella* spp., CFU/g	Not detected	Not detected
Cadmium, mg/kg Cd	<0.010	<0.005
Chromium, mg/kg Cr	<0.010	0.039
Manganese, mg/kg Mn	2.70	3.317
Iron, mg/kg Fe	6.20	11.78
Nichel, mg/kg Ni	0.067	0.286
Lead, mg/kg Pb	<0.010	0.03
Copper, mg/kg Cu	0.78	1.69
Zinc, mg/kg Zn	0.004	0.003
Total N, %	0.29	4.9
Calcium, mg/kg Ca	174.0	407.7
Phosphorus, mg/kg P	196.7	86.8
Magnesium, mg/kg Mg	150.0	316.7
Sodium, mg/kg Na	254.0	57.7
Potassium, mg/kg K	646.0	536.9

No *Salmonella* or moulds were detected in both forages and the *E. coli* load remained below 10 CFU/g. The forage produced with RUW had almost five times the Na content and twice the P content of the reference sample, while the latter had a very high N content. There was no effect of water type on forage height (12.35 vs 12.38 cm for RUW and well water respectively, SEM 0.41, *p* = 0.94) and yield (545 vs 519 kg as fed, SEM 21.5, *p* = 0.41). The nutritional characteristics of HBF and TMR are shown in Tables 4, 5.

**Table 4:** Nutritional characteristics (% of dry matter unless stated) of hydronic barley forage used in the feeding trial.

Item	Hydronic barley forage
Chemical composition	
DM (% of fresh matter)	15.40
Ash	6.49
CP	14.02
NDF	30.97
ADF	17.21
ADL	1.17
Ether extract	3.50
NFC^a^	45.0
Starch	7.14
Total sugar	25.97
NE_L_^b^ (MJ/kg DM)	6.00
Protein fractions (% CP)^c^	
PA	60.61
PB1	5.63
PB2	22.85
PB3	7.13
PC	3.78

a Non-Fibre Carbohydrate calculated as 100 − (%NDF + %CP + %EE + %Ash).

b Net Energy of Lactation, calculated according to INRA equation (39).

c According to the Cornell Net Carbohydrate and Protein System (CNCPS) system version 6.5 (45).

**Table 5:** Ingredients (kg as-fed), chemical composition (% of dry matter unless stated), and particle size distribution (% of dry matter retained of the sieve) of the diets fed to lactating cows.

Item	Control diet^1^	HBF diet^1^
Ingredient		
Sprouted barley	–	10.0
Oat hay	8.0	7.0
Dehydrated alfalfa hay	3.0	3.0
Corn meal	7.5	7.0
Concentrate mix^2^	5.5	5.5
Soybean meal^3^ (44% CP)	2.4	2.4
Molasses	0.4	0.4
Mineral-vitamin premix^4^	0.4	0.4
Water	15.0	7.0
Chemical composition		
DM (% of fresh matter)	56.1	56.0
Ash	9.9	9.9
CP	16.1	16.3
NDF	35.2	34.3
ADF	20.0	19.6
ADL	4.3	4.1
Ether extract	3.78	3.8
NFC^5^	35.1	33.5
Starch	26.2	24.9
Total sugar	3.8	5.4
NE_L_^6^ (MJ/kg DM)	6.7	6.7
Protein fractions (% CP)^7^		
PA	19.2	21.3
PB1	9.6	9.5
PB2	34.1	33.8
PB3	29.9	28.2
PC	7.2	7.1
Particle size distribution (mean ± SD)		
>19.0 mm	49.3 ± 6.0	52.3 ± 7.3
19.0 to 8.0 mm	16.0 ± 2.1	10.2 ± 1.2
<8.0 mm	34.7 ± 4.0	37.6 ± 7.1
Pef^8^	0.65 ± 0.04	0.62 ± 0.07
PeNDF^9^	22.9 ± 0.7	21.4 ± 2.0

^1^ Diets containing (HBF) or not (Control) hydroponic barley forage. ^2^ Contained (g/kg of mix, on a fed basis): 541 g of whole flaked soybean, 196 g of steam-flaked barley, 97 g of whole barley grain, 60 g of calcium carbonate, 50 g of sodium chloride, 34 g of monocalcium phosphate, 22 g of sodium carbonate. ^3^ Solvent extracted. ^4^ Contained (/kg of premix; based on the manufacturer’ declared content): 4.000.000 IU of vitamin A; 100.000 IU of vitamin D3; 1,500 mg of vitamin E; 1,400 mg of vitamin B6; 1,400 mg of vitamin C; 1,100 mg of vitamin B1; 500 mg of vitamin B2; 5,000 mg of choline chloride; 1,000 mg of biotin; 800 mg of pantothenic acid; 700 mg of niacinamide; 180 g calcium; 38 g of phosphorous; 70 g of sodium; 15 g of magnesium; 1,000 mg of S as copper-II-sulphate; 1,600 mg of Mn as manganese-II-oxide; 5,400 mg of Zn as zinc sulphate, monohydrate; 80 mg of I as calcium iodate, anhydrous; 10 mg of Se as sodium selenite. ^5^ Non-Fibre Carbohydrate calculated as 100 − (%NDF + %CP + %EE + %Ash). ^6^ Net Energy of Lactation, calculated according to INRA equation (39). ^7^ According to the Cornell Net Carbohydrate and Protein System (CNCPS) system version 6.5 (45). ^8^ Physical effectiveness factor, determined according to DeVries et al. (49). ^9^ Physically effective NDF, determined according to DeVries et al. (49).

The HBF was characterized by a significant amount of NFC and soluble sugars, a moderate CP content, consisting mainly of non-protein nitrogen and highly soluble protein, and a very low ADF and lignin content. However, the composition of the Control and HBF TMR was very similar.

### 3.2 Clinical, hematological, and biochemical findings

No cows showed clinical signs of active disease during the study. No significant differences were observed between the groups in terms of final BW (660.0 vs 661.7 kg for CG and HBFG, respectively, SEM 12.9, *p* = 0.91) and BCS (3.1 vs 3.2, SEM 0.04, *p* = 0.32). BHB and CBC values remained within the reference range and were not affected by the dietary treatment (Supplementary Table S4), as well as most of the biochemical findings (Table 6).

**Table 6:** Biochemical blood panel (Mean ± SD) of lactating cows fed diets containing (HBFG) or not (CG) hydroponic barley forage on days T-14, T14, T28, and T42.

	Time points				Effect (*P*)		
Parameter	T_−14_	T_14_	T_28_	T_42_	Diet	Time	Diet × Time
AST (55–150 IU/L)					0.6355	0.255	0.6973
CG	122.90 ± 47.29	107.87 ± 59.45	112.83 ± 40.07	106.60 ± 30.90			
HBFG	124.00 ± 51.37	120.10 ± 46.49	116.63 ± 39.47	121.77 ± 40.70			
*p* ^*^	0.92	0.30	0.74	0.19			
ALT (17–37 IU/L)					0.7841	**<0.0001**	0.3036
CG	28.30 ± 8.22	33.53 ± 7.27	36.50 ± 5.89	35.20 ± 7.03			
HBFG	29.43 ± 7.73	33.90 ± 5.43	34.60 ± 5.05	36.53 ± 6.46			
*p* ^*^	0.51	0.83	0.27	0.44			
ALP (29–99 IU/L)					0.4318	**<0.0001**	0.2549
CG	85.53 ± 35.45	58.40 ± 21.63	65.00 ± 29.82	57.60 ± 23.51			
HBFG	78.57 ± 39.83	61.40 ± 25.57	55.60 ± 24.56	50.00 ± 24.93			
*p* ^*^	0.35	0.69	0.21	0.31			
T Bil (0.1–0.6 mg/dL)					0.7590	**<0.0001**	**0.0012**
CG	0.28 ± 0.13	0.07 ± 0.04	0.14 ± 0.04	0.10 ± 0.06			
HBFG	0.31 ± 0.14	0.11 ± 0.06	0.08 ± 0.03	0.07 ± 0.05			
*p* ^*^	0.14	**0.03**	**0.001**	0.12			
TP (5.9–7.7 g/dL)							
CG	7.49 ± 0.45	7.36 ± 0.95	7.63 ± 0.40	7.81 ± 0.58	0.6294	**0.0181**	0.2091
HBFG	7.31 ± 0.67	7.49 ± 1.04	7.79 ± 0.74	7.64 ± 0.60			
*p* ^*^	0.31	0.48	0.37	0.36			
Alb (2.9–3.5 g/dL)					0.2858	0.095	**0.004**
CG	3.47 ± 0.29	3.39 ± 0.38	3.38 ± 0.26	3.54 ± 0.28			
HBFG	3.43 ± 0.32	3.43 ± 0.42	3.63 ± 0.21	3.59 ± 0.22			
*p* ^*^	0.58	0.58	**0.002**	0.50			
Cholesterol (70–170 mg/dL)					0.8960	**<0.0001**	0.3676
CG	150.33 ± 36.56	241.77 ± 59.24	248.37 ± 47.53	238.77 ± 48.95			
HBFG	152.63 ± 53.16	234.33 ± 50.32	256.00 ± 49.08	255.60 ± 45.70			
*p* ^*^	0.86	0.56	0.55	0.19			
Urea (15–35 mg/dL)					0.3527	**<0.0001**	0.4657
CG	33.70 ± 7.11	41.73 ± 8.65	37.13 ± 6.94	35.37 ± 6.59			
HBFG	32.97 ± 8.72	37.73 ± 6.31	36.70 ± 5.95	32.73 ± 5.78			
*p* ^*^	0.70	**0.03**	0.81	0.15			
Cr (0.70–1.10 mg/dL)					0.5834	**<0.0001**	**0.0111**
CG	1.06 ± 0.12	1.05 ± 0.11	0.95 ± 0.14	0.96 ± 0.13			
HBFG	1.04 ± 0.14	1.03 ± 0.16	1.01 ± 0.09	1.05 ± 0.14			
*p* ^*^	0.53	0.44	0.07	**0.01**			
Glu (37–71 mg/dL)					0.8533	**<0.0001**	0.4470
CG	49.57 ± 11.59	63.70 ± 5.51	67.43 ± 4.63	66.73 ± 4.49			
HBFG	50.53 ± 10.11	63.87 ± 8.22	65.23 ± 6.18	68.53 ± 4.42			
*p* ^*^	0.61	0.93	0.25	0.34			
Ca (8.4–10.5 mg/dL)					0.0519	**<0.0001**	0.6845
CG	9.63 ± 0.43	9.25 ± 0.57	9.74 ± 0.34	9.76 ± 0.50			
HBFG	9.55 ± 0.48	9.06 ± 0.90	9.49 ± 0.36	9.68 ± 0.46			
*p* ^*^	0.53	0.15	0.07	0.54			
PHOS (4.5–8.5 mg/dL)					0.5351	**<0.0001**	**0.0375**
CG	6.16 ± 0.86	5.87 ± 0.85	5.58 ± 0.74	5.63 ± 0.79			
HBFG	6.21 ± 1.07	5.52 ± 1.07	5.71 ± 0.76	6.09 ± 0.80			
*p* ^*^	0.82	0.13	0.56	0.04			
Mg (2.0–2.9 mg/dL)					0.0576	**<0.0001**	0.3966
CG	2.39 ± 0.31	2.45 ± 0.30	2.26 ± 0.25	2.74 ± 0.56			
HBFG	*2.41 ± 0.29*	*2.52 ± 0.32*	*2.41 ± 0.24*	*2.66 ± 0.31*			
*p* ^*^	0.82	0.42	0.08	0.36			
Na (136–144 mEq/dL)					0.5150	**<0.0001**	**0.022**
CG	138.33 ± 2.35	134.77 ± 4.37	135.87 ± 3.29	139.47 ± 3.11			
HBFG	137.67 ± 2.37	130.90 ± 12.77	138.43 ± 4.12	134.13 ± 2.78			
*p* ^*^	0.64	**0.007**	0.07	**<0.001**			
K (4.5–5.5 mEq/dL)					0.4817	**0.0032**	0.0835
CG	4.54 ± 0.26	4.27 ± 0.41	4.41 ± 0.29	4.45 ± 0.36			
HBFG	4.49 ± 0.31	4.38 ± 0.50	4.23 ± 0.27	4.38 ± 0.37			
*p* ^*^	0.61	0.23	0.05	0.47			
Cl (90–105 mEq/dL)					0.9329	**<0.0001**	**0.0188**
CG	96.37 ± 2.37	93.13 ± 4.30	94.03 ± 4.20	98.40 ± 2.44			
HBFG	95.80 ± 2.54	90.50 ± 9.90	97.03 ± 5.33	94.73 ± 2.98			
*p* ^*^	0.65	**0.04**	**0.02**	**0.004**			
Ti (57–160 μg/dL)					0.3830	**0.0024**	0.6222
CG	125.20 ± 27.18	127.53 ± 35.85	139.53 ± 27.20	138.40 ± 37.59			
HBFG	118.70 ± 30.87	128.73 ± 28.41	132.17 ± 36.92	143.83 ± 29.13			
*p* ^*^	0.43	0.88	0.37	0.51			
NEFA (0.10–0.50 mEq/L)					0.1549	**<0.0001**	**0.002**
CG	0.20 ± 0.08	0.12 ± 0.04	0.15 ± 0.04	0.12 ± 0.04			
HBFG	0.18 ± 0.07	0.14 ± 0.06	0.11 ± 0.03	0.11 ± 0.08			
*p* ^*^	0.15	0.14	**0.004**	0.72			

Significant *p*-values are printed in bold. Alb, Albumin; ALP, alkaline phosphatase; AST, aspartate aminotransferase; ALT, alanine aminotransferase; Ca, calcium; Cl, chloride; Cr, creatinine; Ti, total iron; Glu, glucose; K, potassium; NA, sodium; Mg, magnesium; NEFA, not esterified fatty acids; PHOS, phosphorus; T Bil, total bilirubin; TP, total proteins. ^*^ Diet effect at each time.

The only exceptions were urea, phosphorus and cholesterol, whose levels were slightly outside the optimal reference range in both CG and HBFG (Table 6). Moreover, against similar initial values (T-14), Cl and Na levels showed an erratic trend, being higher in CG at T14 (*p* = 0.04 and *p* = 0.007, for Cl and Na, respectively) and at T42 (*p* = 0.004 and *p* < 0.001), and in HBFG at T28 (*p* = 0.02 and *p* = 0.07). Finally, the significance of the iterations for T Bil, Alb, Cr, PHOS and NEFA seems to be mainly due to the erratic differences, sometimes only numerical, between the two groups.

### 3.3 Dry matter intake, feed sorting, ruminal pH findings

Dry matter intake tended to be higher (*p* = 0.07) in HBFG group (22.1 vs 23.4 kg/head/d SEM 0.34 for CG vs. HBFG), whereas no differences were observed for diets particle size sorting (Supplementary Table S5). The daily mean pH, the percentage of animals with at least one SARA risk alarm and the number and time of SARA risk alarms detected by the ruminal pH sensor are shown in Table 7.

**Table 7:** Rumen pH (mean ± SD), percentage of animals that presented at least one subacute rumen acidosis (SARA) risk alarm, number and time (min) of SARA risk alarms detected during the experimental period by ruminal pH sensors in lactating cows fed diets containing (HBFG) or not (CG) hydroponic barley forage.

Parameter	Group		
	CG	HBFG	*p* ^^^
Daily mean pH value	6.25 ± 0.28	6.24 ± 0.31	0.76
SARA risk alarm-animals, %	50.0	53.3	0.85 ^*^
SARA risk alarms, *n*	3.13 ± 6.12	5.73 ± 8.67	0.38
Duration of SARA risk alarms, min	1509.67 ± 3061.42	2728.33 ± 4380.27	0.40

^^^ Wilcoxon rank-sum (Mann–Whitney) test. * Test of proportions.

On average, approximately half of the cows (i.e., 16 vs 15 cows for CG and HBFG) in both groups had at least one SARA risk detected. The number and duration of SARA alerts were numerically higher for HBFG, but the differences were far from statistical significance.

### 3.4 Milk production and quality

The milk yield and milk macro components of the GC and HBFG groups are shown in Table 8. The inclusion of HBF from RUW had no effect on the actual milk yield (kg/d) or the percentage of fat, protein, non-fat solid and total solid. Consequently, ECM, fat, and protein production (kg/d) did not differ between the two groups, even though protein production was significantly higher at T1, so that a diet × time interaction was observed (data not shown). Similarly, the higher lactose content at T7 and T42 in HBFG explains the significance of the interaction.

**Table 8:** Milk production (LSM) of cows fed diets containing (HBFG) or not (CG) hydroponic barley forage.

Parameter	Group		SEM	Effect (*p*)		
	CG	HBFG		Diet	Time	Diet × Time
Milk yield kg/d	30.78	30.80	1.104	0.9915	**<0.0001**	0.0508
Energy corrected milk kg/d	30.90	30.73	0.896	0.895	**0.0001**	0.3508
Fat kg/d	1.24	1.23	0.036	0.818	0.0210	0.6341
Protein kg/d	1.12	1.15	0.028	0.5043	**<0.0001**	**0.0237**
Fat %	4.10	4.07	0.113	0.8123	**0.0234**	0.7302
Protein %	3.70	3.80	0.075	0.3476	**<0.0001**	0.6045
Lactose %	4.80	4.88	0.030	0.0512	**0.0029**	**0.0388**
SCC^1^, 10^3^ cells/ mL	164.06	225.18	47.878	0.3705	0.1095	0.1726
Total solid (%)	13.22	13.41	0.176	0.4578	**<0.0001**	0.6204
Non fat solid %	9.15	9.35	0.079	0.0722	**<0.0001**	0.4917
Casein %	2.93	3.02	0.067	0.3267	**<0.0001**	0.7717
Urea mg/dL	30.20	29.77	0.615	0.6286	**<0.0001**	**<0.0001**
pH	6.63	6.65	0.008	0.2372	**<0.0001**	**<0.0001**
Cryoscopy index	−530.77	−534.16	0.758	**0.0025**	**<0.0001**	0.116
*Clotting properties*						
r, min	23.23	26.80	1.287	0.0542	**<0.0001**	**0.0014**
K_20_, min	7.83	7.13	0.253	0.0549	**<0.0001**	**0.0370**
A_30_, mm	29.96	27.79	0.647	**0.0210**	**<0.0001**	**0.0014**

Significant *p*-values are printed in bold. ^1^ Somatic cell count.

Regarding milk constituents and parameters related to technological and hygienic properties, no effects of diet were observed for SCC and casein. On the other hand, the values of cryoscopic index and rennet coagulation time (*r*) were higher (*p* = 0.003) and lower (*p* = 0.04), respectively, in HBFG than in CG. In addition, differences bordering on statistical significance were also observed for curd firming time (K20) (*p* = 0.06) and curd firmness (A30) (*p* = 0.04), which were higher and lower, respectively, in CG. Analogous to protein production and lactose content, the significance of the diet × time iterations found for urea, pH and coagulation properties reflect their irregular trend over the observation period (data not shown). Finally, no significant differences were found between the groups for the FA composition of the milk fat (Supplementary Table S6). No heavy metals were detected in the milk.

### 3.5 Digestibility, nitrogen balance, urine purine derivatives, microbial N supply

No differences between the groups were observed for *in vivo* digestibility (Supplementary Table S7). Table 9 shows the N metabolism parameters.

**Table 9:** Nitrogen balance, urinary excretion of creatinine and purine derivatives (PD), and microbial N supply (LSM) of lactating cows fed diets containing (HBFG) or not (CG) hydroponic barley forage.

Item	Group		SEM	*p*
	CG	HBFG		
N balance, g/d				
N intake	574.82	603.31	8.81	0.04
Faecal N excretion	189.16	191.46	5.45	0.76
Urinary N excretion	182.63	194.27	8.17	0.33
N in milk	186.55	195.01	9.23	0.52
N retained	16.47	22.57	9.08	0.64
Productive N^a^	217.58	223.41	9.33	0.28
Total N excretion^b^	371.80	385.73	9.33	0.30
Urinary excretion				
Urine volume, L/d	26.21	31.96	2.11	0.07
Creatinine, mmol/d	1188.83	1204.24	39.16	0.78
Allontoin, mmol/d	363.52	384.90	10.68	0.17
Uric Acid, mmol/d	63.15	71.79	5.37	0.27
Total PD, mmol/d	426.67	456.69	11.67	0.08
Microbial N supply^c^, g/d	267.93	289.34	8.08	0.07

a Calculated as sum of N in milk and N retained (57).

b Calculated as sum of faecal N and N in urine (57).

c Calculations based on equation from Chen and Gomes (56).

The HBF-fed group had a higher (*p* = 0.04) N intake (+ 39 g/d) due to the higher DMI (+ 1.3 kg/d), together with a higher estimated urine volume (*p* = 0.06). In contrast, the distribution of N in milk, urine and faeces and the urinary excretion of allantoin and uric acid were not affected by the dietary treatments, although a trend towards an increase in estimated MN supply (*p* = 0.07) was observed in the HBFG group.

## 4 Discussion

### 4.1 Reclaimed urban wastewater and HBF yield and quality

The great interest in the use of reclaimed wastewater for agriculture purposes mainly concerns arid and semi-arid regions, but increasing water consumption for irrigation has led to technological innovations and regulatory frameworks for its safe use also in the EU. The criteria set out in Regulation 2020/741 (17) focus on the physico-chemical (e.g., oxygen demand, total suspended solids, turbidity) and microbiological (e.g., *Salmonella* and *E. coli* load) characteristics of the effluent. According to this legislative framework, the RUW used in the present study to produce HBF falls under ‘class B’ and can be used for non-food crops, including those intended for the feeding of dairy or meat livestock. Other irrigation water quality criteria related to human and livestock safety, crop growth, soil protection and irrigation methods (e.g., toxicant levels, pH, electrical conductivity, and SAR) indicated an overall good suitability of RUW (14, 36). There is no regulatory framework for the use of RUW both for HF production and, in general, for hydroponic systems, and the literature on this specific topic is scant and hardly addresses the issue of water quality. Connecting hydroponic systems to urban wastewater treatment plants can provide both a continuous supply of RUW throughout the year and nutrients to the plants, especially nitrogen and phosphorus, which as the main plant nutrients do not pose a risk *per se* in irrigation water (18). However, in contrast to the results of others (32, 34), who observed an improvement in fodder production, the yields of HBF herein described were not different when using RUW or well water. Thus, on the one hand, the use of RUW did not adversely affect the germination and growth processes, as may be the case when high levels of contaminants (i.e., heavy metals and toxic substances and organic pollutants) remain in the effluent (58, 59). On the other hand, the lack of improvement in yield indicates that the extremely short production cycle of HBF did not allow the nutrient supply of the RUW to be exploited. Hypothetically, the high nitrogen content of the HBF from the well water (Table 3) could be indicative of nitrate pollution of the groundwater, which could have masked the differences between the HBF yields. However, it is important to note that photosynthetic activity does not begin until five days after the first root appears, and only then is N required for plant growth (60).

The chemical composition of HBF differs from that of conventionally produced forage due to the very short growth cycle, which does not allow the deposition of cellulose and lignin on the cell wall, and for the presence of not only of the epigeal part (leaves and stems) but also of ungerminated or germinated residue seeds and young roots. These characteristics explain the lower content of lignin and cellulose, the better digestibility of NDF, and the higher content of soluble proteins and sugars compared to conventional forages (61, 62). As a feed, HBF could be compared to maize silage or mash, with the major difference, as already mentioned, that it is a fresh feed. Our results on the composition of HBF are largely in agreement with literature reports (31, 35, 63–66) further confirming that RUW did not interfere with normal seed germination and growth processes during HBF production.

### 4.2 Cow health

The use of RUW to produce HBF can pose health risks to lactating cows, mainly due to the potential presence of microbiological agents. Indeed, hydroponic forage production implies that water comes into contact with the forage, potentially carrying harmful microorganisms. As mentioned above, the EU Regulation aims to significantly reduce this risk by setting strict limits for *Salmonella* and *E. coli* loads, i.e., the main microbial species used for certificate water safety (67). The RUW disinfection with chlorine dioxide used in the present study and as it is commonly used in hydroponic systems to prevent mould growth in a high RH environment (68), provided a further microbiological risk reduction. Indeed, the HBF produced with RUW had no *Salmonella* spp., *E. coli* levels within safe limits [i.e., < 10 CFU (13)], and, considering that the mould index was close to zero during the trial, no relevant mould growth was detected. Urban effluent can contain levels of heavy metals that are hazardous to animal and human health (5, 13). However, these contaminants were at very low or near zero levels in the RUW used and were not detected in the HBF or in the milk samples collected at the end of the trial, i.e., after 7 weeks of daily feeding with the RUW-produced HBF. Compared to the existing literature, these results are of particular interest, because the use of RUW in traditional forage production systems can leads to feed contamination, exposing animals to an unavoidable microbiological risk (67, 69–72) and a dangerous increase in heavy metal intake (73–76).

The HBF-fed cows were in good health and no clinical manifestations of mastitis, laminitis, or other diseases or health and metabolic disorders occurred during the study. In addition to the absence of clinical signs of ongoing disease, indirect but crucial indicators of the health status of the cows such as CBC and key biochemical findings did not show significant variations (77, 78), being within the reference range for healthy cows and not differing substantially between the CG and HBFG groups. The very high Na and Cl contents in both RUW and HBF (Tables 2, 3) may have influenced their irregular serum levels (Table 6) through ionic excretion phenomena typical of the renal tubules (79), although a definitive explanation will require future investigation. Overall, these results support the hypotheses that the use of RUW for HBF production under the conditions tested can be considered safe for the health status of lactating cows. The effects of wastewater-derived feeds on animal metabolism and health have been investigated in a limited number of studies (5, 80). Therefore, this study can be considered one of the first to address the need for a more sustainable use of water in livestock production by recycling this finite and limiting resource. As for the study by Terrè et al. (5), they found that the use of RUW for drinking and preparing milk replacer had no short-term effects on the health and performance of young calves. In contrast, Al-Qudah et al. (80) found symptoms of nitrate poisoning in dairy herds fed fresh grass irrigated with treated municipal water.

### 4.3 Milk performances, dry matter intake, *in vivo* digestibility, and nitrogen balance

The inclusion of RUW-produced HBF in the diet had no significant effect on most of the physiological parameters and production traits measured during the feeding trial. A conservative level of inclusion was used, but it was in line with most studies evaluating the effect of hydroponic forage on milk yield and quality (35, 81–86). The lack of differences between the CG and the HBFG in terms of DMI and feed sorting, as well as *in vivo* digestibility and rumen pH, confirms both the palatability of the HBF and the regularity of the digestive process. The scientific literature and our results agree that HBF is a rich source of vitamins, minerals, bioactive enzymes, soluble sugars, and soluble nitrogen (87, 88). However, in agreement with our results, other work has found no effect of HBF on milk yield or quality (35, 82, 83, 89, 90). These contrasting results could easily be due to the different levels of HF inclusion and/or the different diets used. In particular, the improvement in milk yield seems to be related to the amount of HF included in the ration (30). The lack of significant differences in milk quality is also reported by most previous studies (35, 89, 90). Worth noting, the lack of significant differences for SCC is a non-trivial result, as feeding unhealthy, mouldy, contaminated, etc. feed can lead to increased SCC in milk even in the absence of clinical signs of disease (91, 92). The worsening trend of clotting aptitude in HBFG milk, probably driven by the higher cryoscopic index, is not confirmed in the literature and further research is needed to clarify this finding. However, all values of the rheological parameters were well within the normal range. It is well known that fresh forage can have positive effects on the FA composition of milk fat in terms of increasing PUFA, MUFA, and CLA content (21, 25, 93). The lack of significant effects of HBF that we found is not inconsistent with the literature because, as we have shown in previous work (21), low levels of fresh forage inclusion in the diet are unlikely to significantly alter milk fatty acid composition.

The lack of differences for *in vitro* digestibility is in agreement with the observation of others for similar diets (94, 95). For both groups, the level of N excretion was within the range reported by Spanghero and Kowalski (57) for similar N intake, while the NB estimate was lower, probably due to the higher milk N recorded in our study or a combination of unaccounted for N, such as urinary N excretion in forms not detected by the Kjeldhal method (e.g., nitrate), volatile losses of gaseous N and ammonia, and dermal scurf (57, 96). Although the N intake was higher for HBFG, no differences between the groups were observed for N excretion, in contrast with a large body of literature reporting a close relationship between N intake and N urinary excretion (97–101). This finding could be due to a more efficient utilization of ruminal ammonia in HBFG cows resulting in lower portal ammonia uptake and consequently lower urinary N (102). The tendentially higher total PD recovered in urine together with the lack of differences for urea concentration in milk and plasma can indirectly supports this hypothesis. The higher urine production of cows fed the HBF-containing diet probably reflects the higher intake of Na, along with other minerals and trace elements, resulting in increased urine production to maintain optimal plasma osmolarity (103, 104).

## 5 Conclusion

Water and land use associated with livestock production could be reduced by changing feed production methods and using additional water sources, such as wastewater from urban areas. In this study we investigated the effects of using tertiary urban wastewater for hydroponic barley forage production on forage quality, lactating cow health and performance, and milk quality. Wastewater treatment using membrane bioreactor technology was found to be effective in removing bacteria and nematode eggs, while on-farm disinfection of RUW further contributed to maintaining the coliform load at the level set by EU Regulation 741/2020 for irrigation water intended for crops fed to dairy or beef cattle. RUW showed good overall quality as irrigation quality and a fair N and P content as fertilizers, which, however, did not affect the yield and quality of HBF. The characteristics of HBF reflected the quality of the RUW delivered into the growth chambers. The feeding of lactating cows with RUW-derived HBF had no major positive or negative effects on animal health and production, including milk quality, *in vivo* digestibility, and nitrogen balance. It follows that the use of RUW under the conditions considered appears to be safe for the health status of lactating cows and the quality of the milk obtained. Overall, the results do not indicate any major limitations to the use of tertiary wastewater to produce hydroponic barley forage. Therefore, a wider application of RUW as irrigation water in hydroponic systems seems realistic. Future studies that take into account the accurate measurement of RUW consumption, characteristics and utilization of RUW effluent from hydroponic chamber along with the evaluation of the impact of higher feeding levels of RUW-produced HBF on animal health and performance, i.e., the main limitations of this study, will be of interest to fully validate these outcomes and provide a more comprehensive indication of the potential of the two coupled technologies to improve the environmental sustainability of animal agriculture. Furthermore, even if the quality of RUW is certified by the treatment plant, its use still requires on-farm monitoring of crops and the health status of lactating cows, as well as the adoption of appropriate hygiene practices.

Finally, it should be stressed that the economic aspect of HBF production cannot be considered “negligible” in the decision to use this type of cultivation, although it does support the objectives of this paper. Despite the possibility of reusing a water resource such as wastewater, hydroponic cultivation required significant economic investment and variable costs, mainly related to the purchase of growth chambers and barley seeds, which could affect the economic sustainability of this type of production. Further technological and design studies are needed to develop growth chambers that increase productivity and reduce costs.

## Data availability statement

The raw data supporting the conclusions of this article will be made available by the authors, without undue reservation.

## Ethics statement

The animal studies were approved by the Institutional Review Board of the Italian Ministry of Health (protocol code 74371-X/10, no. 1,031/2020-PR, Date of approval 19, November 2021). The studies were conducted in accordance with the local legislation and institutional requirements. Written informed consent was obtained from the owners for the participation of their animals in this study.

## Author contributions

LC: Writing – original draft, Conceptualization, Funding acquisition. MC: Writing – original draft, Data curation, Investigation. FS: Data curation, Writing – original draft, Writing – review & editing. AF: Conceptualization, Investigation, Data curation, Writing – review & editing, Formal analysis, Supervision. FM: Writing – original draft, Writing – review & editing. GC: Funding acquisition, Project administration, Conceptualization, Writing – review & editing.
