# Supplementary material for: Use of reclaimed urban wastewater for the production of hydroponic barley forage: water characteristics, feed quality and effects on health status and production of lactating cows
Source: Front Vet Sci. 2023 Nov 17;10:1274466. doi: 10.3389/fvets.2023.1274466 (PMC10690813; doi:10.3389/fvets.2023.1274466)
Supplement: Supplementary file 1 [file Data_Sheet_1.pdf]

Table S1. Determination methods of the microbiological, physical, and chemical parameters of the reclaimed urban wastewater

**Parameter**	**Method**
Turbidity, NTU	APAT CNR IRSA 2110 Man 29 2003
Biochemical oxygen demand (BOD), mg O_2/L	ISO 5815:1-2019
Total suspended solids, mg/L	APAT CNR IRSA 2090 B Man 29 2003
Escherichia coli, CFU/100 mL	APAT CNR IRSA 7030 D Man 29 2003
Intestinal nematodes, eggs/L	Optical microscope observation
Salmonella spp, CFU/g	ISO 19250:2010
pH	APAT CNR IRSA 2060 Man 29 2003
Electrical conductivity at 20°C, µS/cm	APAT CNR IRSA 2030 Man 29 2003
Total dissolved solids 180 °C, mg/L	APAT CNR IRSA 2090 A Man 29 2003
Sodium adsorption ratio (SAR), meq/L	DM 23/03/2000 GU n 87 13/04/2000 SO n 60 All 1 pp 20-21
Ammonium Nitrogen, mg/L N-NH_4	APAT CNR IRSA 4030 C Man 29 2003
Nitrate Nitrogen, mg/L N-NO_3	APAT CNR IRSA 4020 C Man 29 2003
Total Nitrogen, mg/L N	APAT CNR IRSA 4060 C Man 29 2003
Phosphorus, mg/L P	APAT CNR IRSA 4060 C Man 29 2003
Chlorine, mg/L Cl	APAT CNR IRSA 4020 C Man 29 2003
Fluoride, mg/L F	APAT CNR IRSA 4020 C Man 29 2003
Sodium, mg/L Na	APAT CNR IRSA 3030 C Man 29 2003
Potassium, mg/L K	APAT CNR IRSA 3030 C Man 29 2003
Magnesium, mg/L Mg	APAT CNR IRSA 3030 C Man 29 2003
Calcium, mg/L Ca	APAT CNR IRSA 3030 C Man 29 2003
Aluminium, µg/L Al	ISO 15587-1:2002+UNI ISO 17294-2:2016
Arsenic, µg /L As	ISO 15587-1:2002+UNI ISO 17294-2:2016
Boron, µg /L B	ISO 15587-1:2002+UNI ISO 17294-2:2016
Cadmium, µg /L Cd	ISO 15587-1:2002+UNI ISO 17294-2:2016
Chromium, µg /L Cr	ISO 15587-1:2002+UNI ISO 17294-2:2016
Iron, µg /L Fe	ISO 15587-1:2002+UNI ISO 17294-2:2016
Manganese, µg /L Mn	ISO 15587-1:2002+UNI ISO 17294-2:2016
Mercury, µg /L Hg	ISO 15587-1:2002+UNI ISO 17294-2:2016
Nickel, µg /L Ni	ISO 15587-1:2002+UNI ISO 17294-2:2016
Lead, µg /L Pb	ISO 15587-1:2002+UNI ISO 17294-2:2016
Copper, µg /L Cu	ISO 15587-1:2002+UNI ISO 17294-2:2016
Vanadium, µg /L Va	ISO 15587-1:2002+UNI ISO 17294-2:2016
Zinc, µg /L Zn	ISO 15587-1:2002+UNI ISO 17294-2:2016

Table S2. Determination methods of the microbiological load, content of metals and non-metals of the hydroponic barley forage

**Parameter**	**Method**
Escherichia coli, CFU/g	ISO 16649-2:2001
Salmonella spp, CFU/g	AFNOR BIO 12/32-10/11
Cadmium, mg/kg Cd	UNI EN 13804:2013 + UNI EN 13805:2014 + UNI EN ISO 17294-2:2016
Chromium, mg/kg Cr	UNI EN 13804:2013 + UNI EN 13805:2014 + UNI EN ISO 17294-2:2016
Manganese, mg/kg Mn	UNI EN 13804:2013 + UNI EN 13805:2014 + UNI EN ISO 17294-2:2016
Iron, mg/kg Fe	UNI EN 13804:2013 + UNI EN 13805:2014 + UNI EN ISO 17294-2:2016
Nichel, mg/kg Ni	UNI EN 13804:2013 + UNI EN 13805:2014 + UNI EN ISO 17294-2:2016
Lead, mg/kg Pb	UNI EN 13804:2013 + UNI EN 13805:2014 + UNI EN ISO 17294-2:2016
Copper, mg/kg Cu	UNI EN 13804:2013 + UNI EN 13805:2014 + UNI EN ISO 17294-2:2016
Zinc, mg/kg Zn	UNI EN 13804:2013 + UNI EN 13805:2014 + UNI EN ISO 17294-2:2016
Total N, %	ISTISAN 1996/34 p. 124
Calcium, mg/kg Ca	UNI EN 13804:2013 + UNI EN 13805:2014 + UNI EN ISO 17294-2:2016
Phosphorus, mg/kg P	ISTISAN 1996/34 p. 124
Magnesium mg/kg Mg	UNI EN 13804:2013 + UNI EN 13805:2014 + UNI EN ISO 17294-2:2016
Sodium, mg/kg Na	Flame Emission Photometry Method
Potassium, mg/kg K	UNI EN 13804:2013 + UNI EN 13805:2014 + UNI EN ISO 17294-2:2016

Table S3. Distribution of breed, milk yield, days in milk, lactation number, body weight and body condition score (BCS) in cows included in group fed diets containing (HBFG) or not (CG) hydroponic barley forage

**Item**	**CG** (n=30)	**HBFG** (n=30)	**p ¥**
Breed[1]			0.76 ^
Mixed	3 (10.00)	4 (13.33)	
Swiss Brown	9 (30.00)	11 (36.67)	
Holstein Friesian	18 (60.00)	15 (50.00)	
Milk yield[2], kg/d	30.75±9.58	29.33±10.08	0.33
Day in milk[2], d	140.64±87.10	161.52±95.80	0.49
Lactation number[2]	1.90±0.96	1.90±1.24	0.63
Body Weight, kg[2]	651.50±69.69	635.77±89.54	0.36
BCS	2.82±0.25	2.86±0.24	0.41

[1] The values in brackets represent the percentage of each breed on the total of cows within the groups.

[2] As mean and standard deviation.

[¥] Wilcoxon rank-sum (Mann–Whitney) test.

[^] Fisher's exact test.

Table S4. Complete Blood Count parameters (Mean ± SD) of lactating cows fed diets containing (HBFG) or not (CG) hydroponic barley forage on days T-14, T14, T28, and T42

Parameter[1]	Time points				Effect (*P*)		
	T_{-14}	T_{14}	T_{28}	T_{42}	Diet	Time	Diet x Time
RBC (5-9.99 ×106/µl)					0.64	**<0.0001**	0.14
CG	6.0±0.143	6.75±0.143	6.90±0.143	6.76±0.143			
HBFG	6.1±0.143	6.78±0.143	6.66±0.143	6.60±0.143			
P *	*0.86*	*0.90*	*0.24*	*0.43*			
HGB (8-15 g/dl)					0.52	**<0.0001**	0.36
CG	10.13±0.203	11.13±0.203	11.47±0.203	11.11±0.203			
HBFG	10.01±0.203	11.47±0.203	11.14±0.203	10.84±0.203			
P *	*0.69*	*0.25*	*0.26*	*0.36*			
HCT (24-46 %)					0.30	**<0.0001**	0.62
CG	26.10±0.442	28.58±0.442	29.29±0.442	28.99±0.442			
HBFG	25.80±0.442	28.91±0.442	28.49±0.442	28.45±0.442			
P *	*0.64*	*0.60*	*0.20*	*0.39*			
WBC (4-12 ×10^3/µl)					0.155	**0.0248**	0.42
CG	9.17±0.316	8.96±0.316	8.50±0.316	9.00±0.316			
HBFG	9.38±0.316	8.64±0.316	8.18±0.316	8.16±0.316			
P *	*0.64*	*0.48*	*0.47*	*0.06*			
NEU (0.6-4.0 /µl)					**0.07**	0.53	0.40
CG	4.15±0.253	4.11±0.253	3.89±0.253	4.28±0.253			
HBFG	4.13±0.253	3.85±0.253	3.69±0.253	3.44±0.253			
P *	*0.96*	*0.46*	*0.58*	***0.02***			
LYM (2.5-7.5 /µl)					0.39	**0.03**	0.56
CG	3.66±0.190	3.59±0.190	3.32±0.190	3.45±0.190			
HBFG	4.06±0.190	3.53±0.190	3.25±0.190	3.63±0.190			
P *	*0.14*	*0.84*	*0.80*	*0.51*			
MONO (0.0-8.0 /µl)					0.30	**0.02**	0.67
CG	0.91±0.04	0.85±0.04	0.81±0.04	0.88±0.04			

HBFG	0.93±0.04	0.81±0.04	0.78±0.04	0.81±0.04			
P *	*0.62*	*0.49*	*0.52*	*0.23*			
EOS (0.0-2.4/µl)					0.09	**0.009**	0.17
CG	0.28±0.06	0.41±0.31	0.56±0.54	0.38±0.29			
HBFG	0.25±0.23	0.44±0.37	0.44±0.21	0.28±0.18			
P *	***0.04***	*0.99*	*0.98*	*0.99*			
PLT (100-800 K/µl)					0.91	**0.013**	0.87
CG	342.57±20.30	301.31±20.30	317.33±20.30	301.80±20.30			
HBFG	356.33±20.30	299.24±20.30	305.97±20.30	309.90±20.30			
P *	*0.63*	*0.94*	*0.69*	*0.78*			

Significant P-values are printed in bold.

* *Diet effect at each time.*

[1]RBC, Red blood cells; HGB, Hemoglobin; HCT, Hematocrit; WBC, White blood cells; NEU, Neutrophils; LYM, Lymphocytes; MONO, Monocytes; EOS, Eosinophils; PLT, Platelets.

Table S5. Dry matter intake (DMI), and sorting behaviour of the long, medium, and fine particles[1] (LSM) of lactating cows fed diets containing (HBFG) or not (CG) hydroponic barley forage

Item	Group		SEM	*P*
	CG	HBFG		
Overall DMI, kg/d	22.12	23.38	0.34	0.07
Predicted intake[2], kg/d				
Long	11.17	12.88	0.82	0.36
Medium	3.61	2.48	0.21	0.02
Fine	7.85	9.15	0.43	0.20
Actual intake[3], kg/d				
Long	10.92	12.31	0.81	0.46
Medium	3.54	2.32	0.17	0.02
Fine	7.66	8.75	0.35	0.52
Sorting index[4], %				
Long	100.20	99.74	0.85	0.79
Medium	99.83	98.14	2.20	0.62
Fine	99.78	100.18	1.26	0.83

[1] Particle size determined by a Penn State Particle Separator, which has a 19-mm screen (long), an 8-mm screen (medium), pan (fine, <8 mm).

[2] The predicted intake of each fraction was calculated as DMI of the complete ration multiplied by the percentage of the fraction in the offered ration (on a DM basis).

[3] The actual intake of each fraction was calculated as the difference between the amount of each fraction in the offered feed and that in the refused feed (on DM basis).

[4] The sorting index (SI) was calculated as 100 x (n actual DMI/ n predicted DMI), where n= particle fraction by a Penn State Particle Separator. Sorting values equal to 100% indicate no sorting, > 100% indicate a preferential consumption (sorting for), and < 100% indicate a selective refusal (sorting against).

Table S6. Fatty acids composition (% of total FA; LSM) of milk fat of lactating cows fed diets containing (HBFG) or not (CG) hydroponic barley forage

**Fatty acid**	**Group**		**SEM**	***P***
	**CG**	**HBFG**		
C4:0	4.03	4.53	0.186	0.07
C6:0	2.51	3.27	0.35	0.14
C8:0	1.28	1.55	0.186	0.33
C10:0	1.93	1.51	0.30	0.34
C11:0	0.24	0.21	0.03	0.47
C12:0	2.68	2.12	0.18	**0.04**
C13:0	0.17	0.18	0.01	0.66
C14:0	10.94	11.42	0.27	0.22
C14:1	0.93	1.05	0.101	0.40
C15:0	1.12	1.27	0.05	0.07
C16:0	36.1	36.5	0.88	0.77
C16: 1	1.61	2.08	0.15	**0.03**
C17:0	0.50	0.43	0.04	0.21
C17:1	0.18	0.23	0.04	0.37
C18:0	9.56	8.34	0.36	0.24
C18:1*trans*-11	0.23	0.25	0.01	0.65
C18:1*cis*-9	21.5	20.4	0.79	0.36
C18:2*cis*9,12	2.68	2.42	0.11	0.11
C18:2*trans*9,12	0.31	0.27	0.02	0.27
C20:0	0.24	0.22	0.02	0.49
C18:3 n-3	0.38	0.35	0.04	0.65
C18:2 cis-9,*trans*-11	0.44	0.43	0.04	0.84
C22:0	0.24	0.21	0.02	0.32
C20:3	0.19	0.15	0.02	0.10
Saturated	71.5	73.3	0.98	0.59
MUFA	24.4	24.0	0.90	0.76
PUFA	4.01	3.63	0.16	0.11

Table S7. In vivo digestibility coefficients (LSM) of lactating cows fed diets containing (HBFG) or not (CG) hydroponic barley forage

**Item**	**Group**		**SEM**	***P***
	**CG**	**HBFG**		
Dry matter	63.82	63.33	0.75	0.65
Organic matter	64.71	65.66	0.71	0.36
Crude Protein	66.37	67.25	0.73	0.41
NDF	33.94	30.86	1.67	0.21
ADF	30.14	28.21	1.34	0.32

NDF, neutral detergent fibre; ADF, acid detergent fibre.
